# Publisher Correction: Attractive and repulsive effects of sensory history concurrently shape visual perception

**DOI:** 10.1186/s12915-022-01493-y

**Published:** 2022-12-08

**Authors:** Jongmin Moon, Oh-Sang Kwon

**Affiliations:** Department of Biomedical Engineering, Ulsan National Institute of Science and Technology, 50 UNIST-gil, Ulsan, 44919 South Korea

**Correction: BMC Biol 20, 247 (2022)**

**https://doi.org/10.1186/s12915-022-01444-7**

Following the publication of original article [1], an error introduced at proofing stage was identified by the authors in Eq. (8). The corrected version should read as follows:

$${\displaystyle \begin{array}{c}{\mu}_{\beta}\sim \textrm{Normal}\left(0.5,{0.5}^2\right)\\ {}{\mu}_a\sim \textrm{Uniform}\left(-\infty, \infty \right)\\ {}{\mu}_l\sim \textrm{Uniform}\left(5,90\right)\\ {}{\mu}_{\sigma}\sim \textrm{Uniform}\left(0,\infty \right)\end{array}}$$

The original article [1] has been updated, and we apologise for any inconvenience caused by the error.
