# Supplementary material for: Attractive and repulsive effects of sensory history concurrently shape visual perception
Source: BMC Biol. 2022 Nov 7;20:247. doi: 10.1186/s12915-022-01444-7 (PMC9641899; doi:10.1186/s12915-022-01444-7)
Supplement: Supplementary file 1 — Additional file 1: Figures S1–S7. Figure S1. Idiosyncratic bias as a function of current stimulus direction. Figure S2. Marginal bias plot for representative subjects. Figure S3. Artifactual sequential effect of previous response with and without the bias correction, after shuffling the order of the trials. Figure S4. Individual behavior and distribution of trials. Figure S5. Residuals of the Response model and the Stimulus model. Figure S6. Minimal group difference in model parameters. Figure S7. Results without applying the bias correction. [file 12915_2022_1444_MOESM1_ESM.pdf]

Additional file 1: Figures S1–S7

Attractive and repulsive effects of sensory history concurrently shape visual perception

Jongmin Moon & Oh-Sang Kwon

Department of Biomedical Engineering, Ulsan National Institute of Science and Technology, Ulsan 44919, South Korea

Corresponding author
Oh-Sang Kwon, Ph.D.
Associate Professor, Department of Biomedical Engineering
Ulsan National Institute of Science and Technology, 50 UNIST-gil, Ulsan 44919, South Korea
Email: oskwon@unist.ac.kr

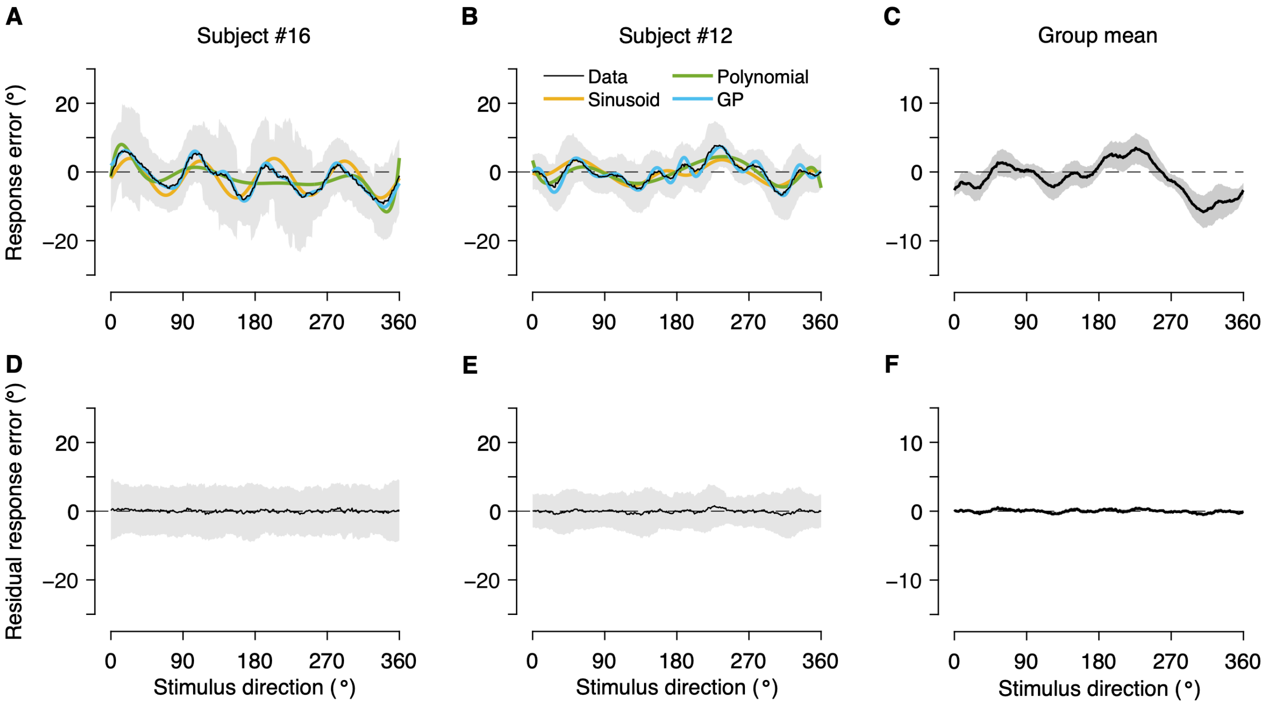

Figure S1. Idiosyncratic bias as a function of current stimulus direction. (**A**) Direction estimation bias as a function of current stimulus direction in raw responses for an individual subject. Bias was computed by averaging the trials over a sliding window (window width: 22.5°, step size: 1°). For this subject, responses are strongly biased away from the cardinal axes, and the bias function takes a sinusoidal form with its period approximately equal to 90°. (**B**) Same as in **A**, but for a different subject. For this subject, the bias function cannot be easily described as bias toward or away from the cardinal axes. (**C**) Mean direction estimation bias as a function of current stimulus direction. (**D**–**F**) Same as in **A**–**C**, but in residualized responses (i.e., after applying the bias correction). For **A**, **B**, **D**, and **E**, shaded regions represent ±1 SD; for **C** and **F**, shaded regions represent 95% confidence interval.

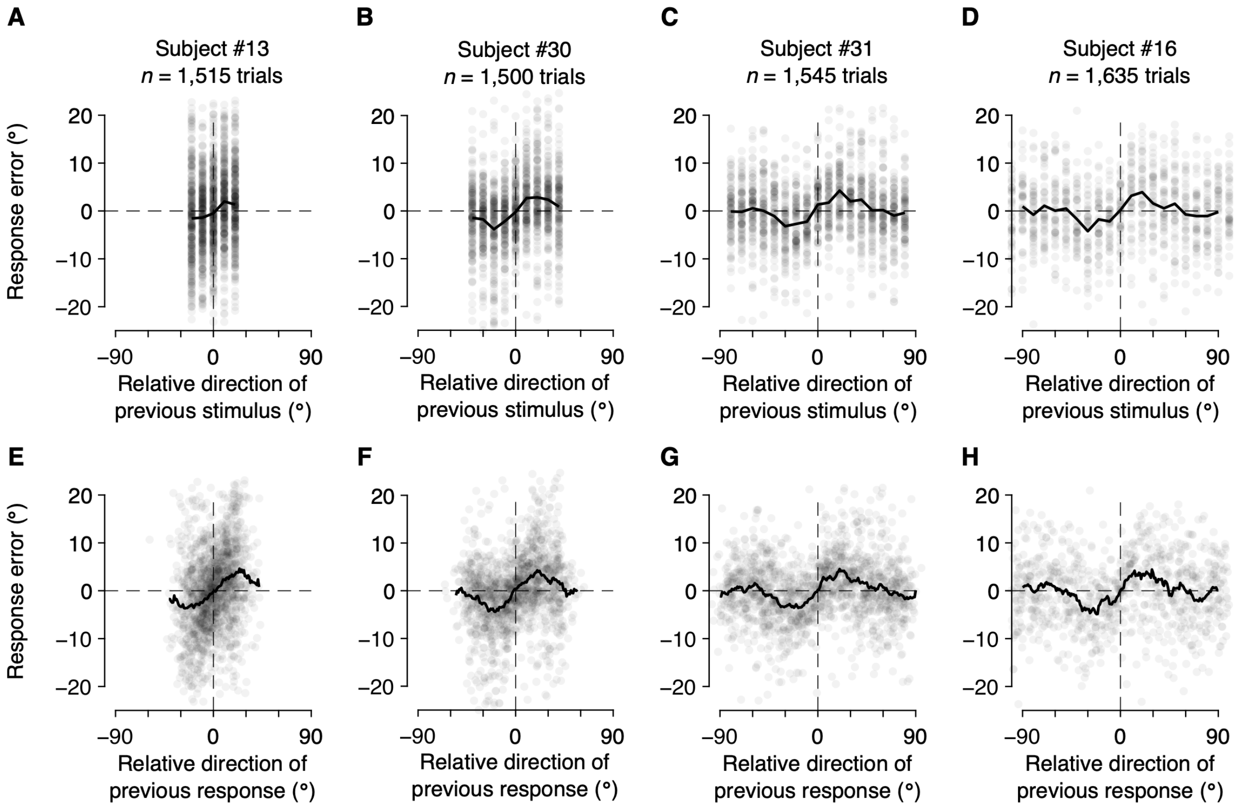

Figure S2. Marginal bias plot for representative subjects. (**A**–**D**) Same as in **Figure 1B**, but for representative subjects in the ±20°, ±40°, ±90°, and ±180° group, respectively. Semi-transparent dots represent response errors on each trial. (**E**–**H**) Same as in **Figure 1C**, but for the representative subjects in **A**–**D**.

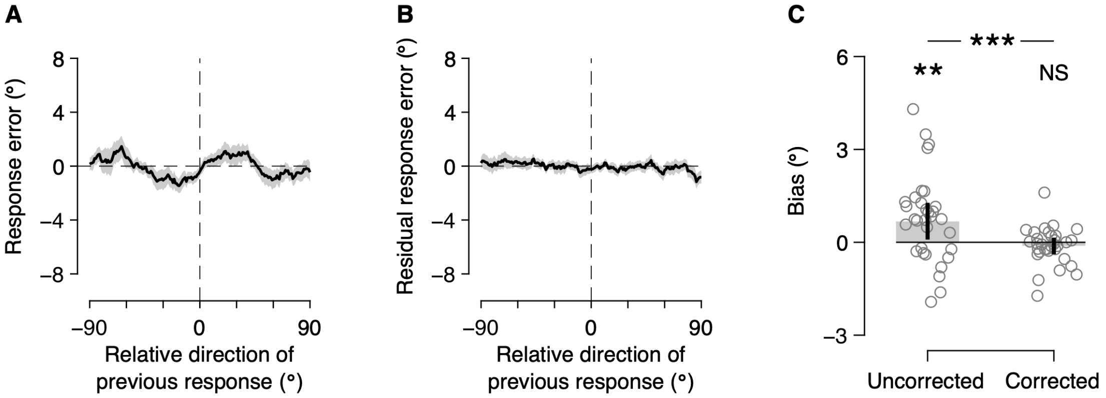

Figure S3. Artifactual sequential effect of previous response with and without the bias correction, after shuffling the order of the trials. (**A**) Same as in **Figure 1C**, but after shuffling the order of the trials and not applying the bias correction. Even after we shuffled the order of the trials, there is still artifactual sequential effect of previous response. Shaded regions represent 95% confidence interval. (**B**) Same as in **A**, but after applying the bias correction. Our correction procedure successfully removed the confounding bias, such that no artifactual sequential effect emerged when we shuffled the order of the trials. (**C**) The Response model fit to the temporally shuffled datasets with and without applying the bias correction. Bars represent the posterior estimate of the population mean, error bars represent 95% credible interval, and open circles represent individual parameter estimates. ***$P < 0.001$, **$P < 0.01$, NS $P > 0.05$.

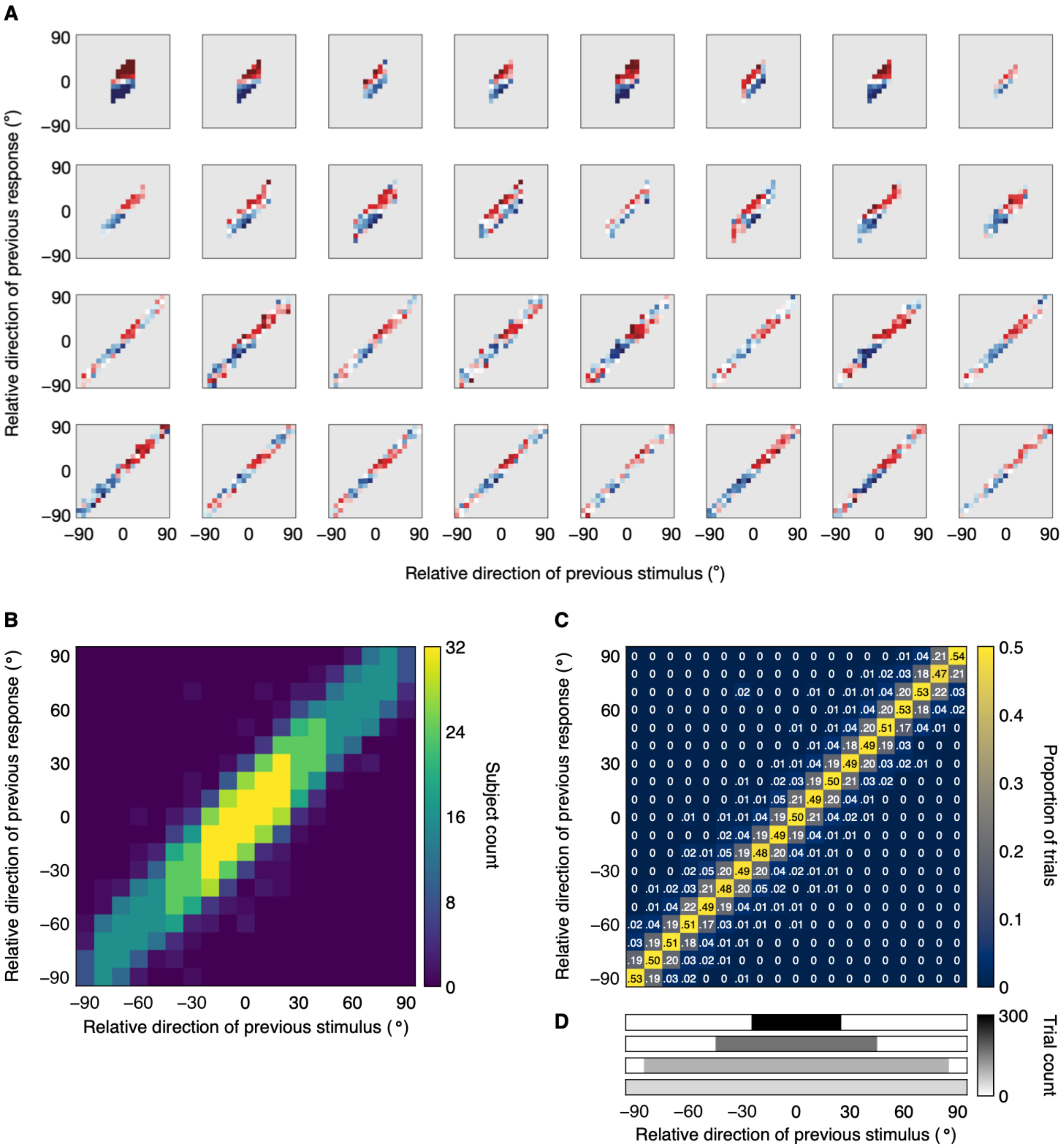

Figure S4. Individual behavior and distribution of trials. (**A**) Joint bias map for individual subjects. Color conventions are as described in **Figure 2A**, and gray regions indicate bins with less than five trials. (**B**) Subject count for each pixel in **Figure 2A**. (**C**) Mean trial count for each pixel in **Figure 2A**, normalized by the number of trials each subject completed for each relative direction of previous stimulus (i.e., x-axis value). (**D**) Marginal distribution of trials for the ±20°, ±40°, ±80°, and ±180° group, from top to bottom, respectively. Multiplying a proportion of trials shown in **C** with the corresponding trial count shown in **D** gives the average number of trials in the pixel for an individual subject in each group.

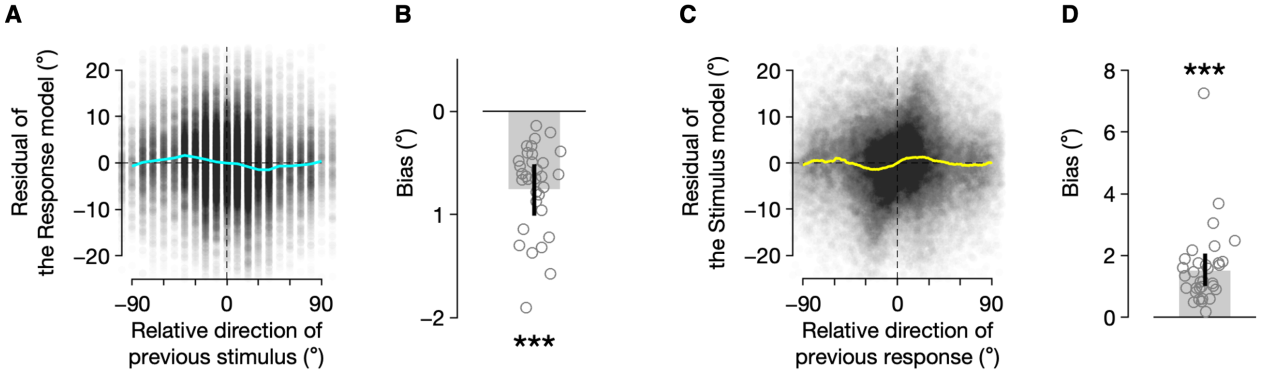

Figure S5. Residuals of the Response model and the Stimulus model. (**A**) Residuals of the Response model as a function of the relative direction of the previous stimulus. Semi-transparent dots represent residuals on all trials pooled from all subjects, and solid line represent moving averages of the pooled data (window width: 10°, step size: 1°). (**B**) The Stimulus model fit to residuals of the Response model. Bar represents the posterior estimate of the population mean, error bar represents 95% credible interval, and open circles represent individual parameter estimates. The repulsive sequential effect of the previous stimulus was highly significant (–0.75° [–1.01 –0.52], $t_{(31)} = 9.922$, $P < 0.001$). (**C**) Residuals of the Stimulus model as a function of the relative direction of the previous response. (**D**) The Response model fit to residuals of the Stimulus model. The attractive sequential effect of the previous response was highly significant (1.51° [1.00 2.07], $t_{(31)} = 6.894$, $P < 0.001$). ***$P < 0.001$

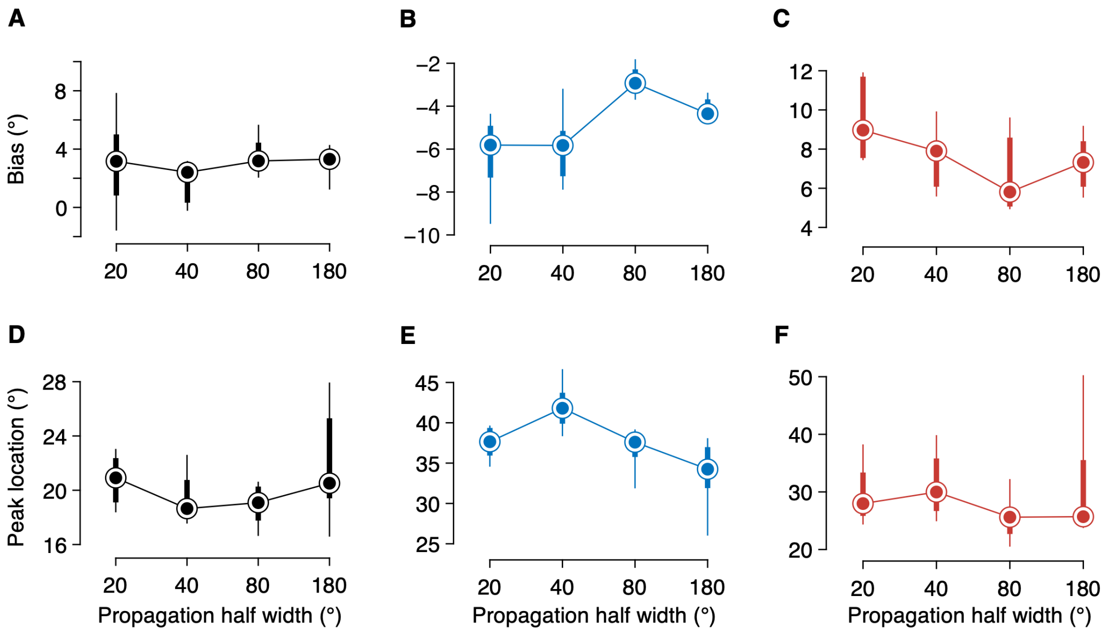

Figure S6. Minimal group difference in model parameters. (**A**) Group difference in bias magnitudes estimated by the Stimulus model. There was no significant effect of propagation noise (P = 0.157; Kruskal–Wallis test). (**B**) Group difference in magnitudes of bias away from the previous stimulus, estimated by the stimulus & Response model. There was a significant main effect of propagation noise on the bias magnitude (P < 0.001). A post hoc Tukey test showed that only the 80° group had significantly weaker repulsive biases compared to the 20° group (P = 0.002) and the 40° group (P = 0.002). (**C**) Group difference in magnitudes of bias toward the previous response, estimated by the Stimulus & Response model. No statistical significance was found (P = 0.125). (**D**–**F**) Same as in **A**–**C** but for group differences in locations of maximum biases. There was a significant main effect of propagation noise on the peak location of the repulsive bias estimated by the Stimulus & Response model (P = 0.032). A post hoc Tukey test showed that only the 40° group had significantly widely tuned repulsive biases compared to the 180° group (P = 0.018). No statistical significance was found in peak locations estimated by the Stimulus model (P = 0.230) and peak locations of attractive biases estimated by the Stimulus & Response model (P = 0.199). Circles represent the median, filled boxes represent the interquartile range, and whiskers represent the 1.5 times the interquartile range away from the 25th and 75th percentiles. Outliers beyond the whiskers are omitted for illustration.

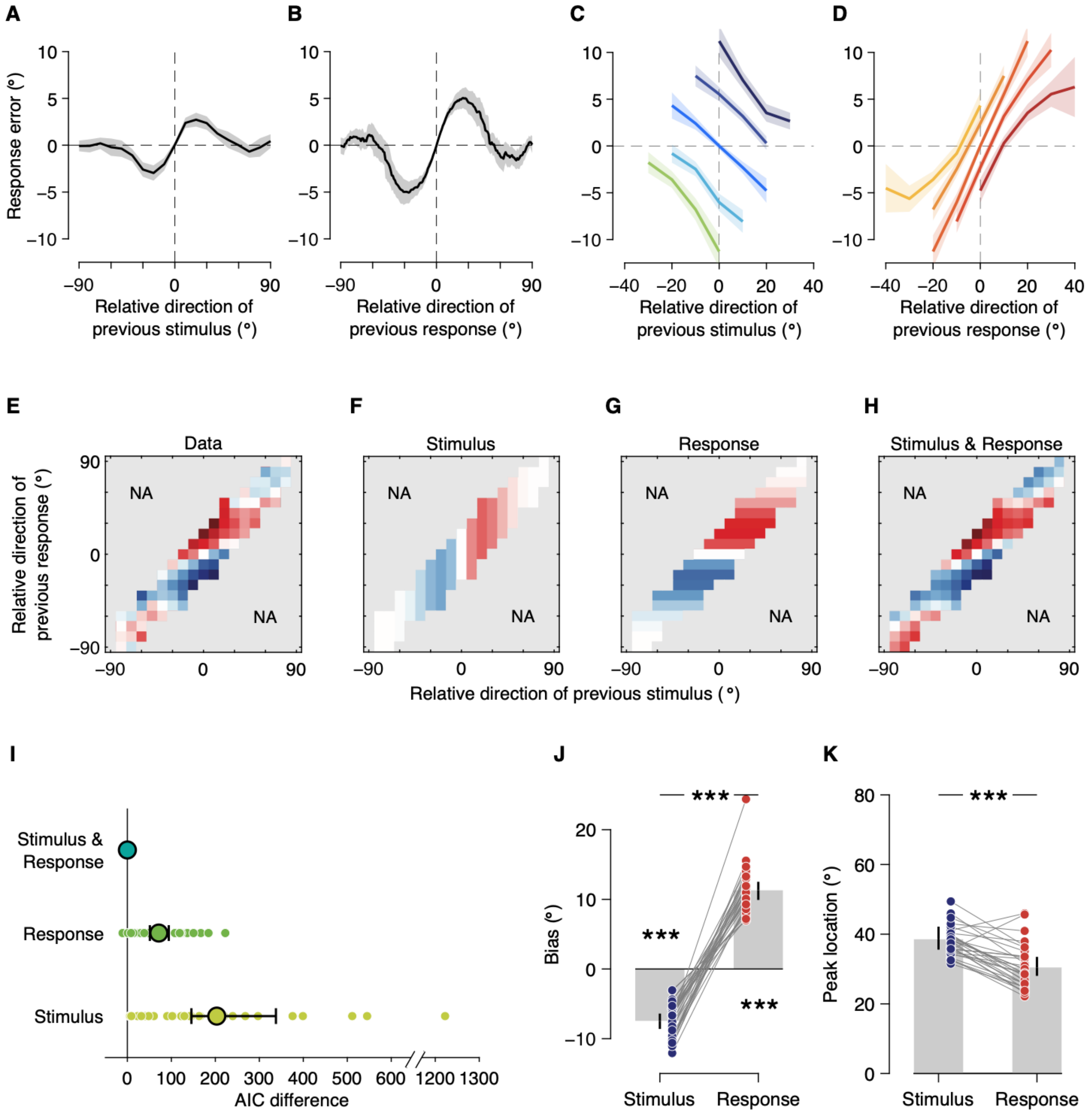

Figure S7. Results without applying the bias correction. (**A**) Same as in **Figure 1B**, but without applying the bias correction. Amplitude = 3.00° [2.27 3.79], $t_{(31)}$ = 8.848, $P < 0.001$. (**B**) Same as in **Figure 1C**, but without applying the bias correction. Amplitude = 5.31° [4.07 6.58], $t_{(31)}$ = 8.882, $P < 0.001$. (**C**, **D**) Same as in **Figure 2B** and **2C**, but without applying the bias correction. (**E**) Same as in **Figure 2A**, but without applying the bias correction. (**F**–**I**) Same as in **Figure 3**, but without applying the bias correction. (**J**) Same as in **Figure 4A**, but without applying the bias correction. Previous stimulus: –7.44° [–8.61 –6.40], $t_{(31)}$ = 17.487, $P < 0.001$; previous response: 11.30° [9.92 12.52], $t_{(31)}$ = 19.190, $P < 0.001$; difference in absolute magnitude: 3.72° [2.12 5.35], $t_{(31)}$ = 22.468, $P < 0.001$. (**K**) Same as in **Figure 4B**, but without applying the bias correction. Previous stimulus: 38.54° [35.57 42.16]; previous response: 30.44° [28.03 33.48]; difference: 7.86° [4.04 12.06], $t_{(31)}$ = 7.596, $P <$ 0.001.
